# Differences of respiratory mechanics in mechanical ventilation of acute respiratory distress syndrome between patients with COVID-19 and Influenza A

**DOI:** 10.1186/s12931-024-02730-4

**Published:** 2024-03-07

**Authors:** Eunki Chung, Ah Young Leem, Kyung Soo Chung, Young Ae Kang, Moo Suk Park, Young Sam Kim, Hye Jin Jang, Su Hwan Lee

**Affiliations:** 1 Division of Pulmonology, Department of Internal Medicine, National Health Insurance Service Ilsan Hospital, Goyang, Republic of Korea; 2 Division of Pulmonary and Critical Care Medicine, Department of Internal Medicine, Severance Hospital, Yonsei University College of Medicine, 50-1 Yonsei-ro, Seodaemun-gu, Seoul, 03722 Republic of Korea; 3 Division of Pulmonary, Department of Internal Medicine, Inha University Hospital, Inha University College of Medicine, 27, Inhang-Ro, Jung-Gu, Inchon, 22332 Republic of Korea

**Keywords:** Acute respiratory distress syndrome, COVID-19, Influenza A, Mechanical ventilation

## Abstract

**Background:**

Whether COVID-19-induced acute respiratory distress syndrome (ARDS) should be approached differently in terms of mechanical ventilation therapy compared to other virus-induced ARDS is debatable. Therefore, we aimed to ascertain whether the respiratory mechanical characteristics of COVID-19-induced ARDS differ from those of influenza A induced ARDS, in order to establish a rationale for mechanical ventilation therapy in COVID-19-induced ARDS.

**Methods:**

This was a retrospective cohort study comparing patients with COVID-19-induced ARDS and influenza A induced ARDS. We included intensive care unit (ICU) patients with COVID-19 or Influenza A aged ≥ 19, who were diagnosed with ARDS according to the Berlin definition between January 2015 and July 2021. Ventilation parameters for respiratory mechanics were collected at specific times on days one, three, and seven after intubation.

**Results:**

The median age of the 87 participants was 71.0 (62.0–78.0) years old, and 63.2% were male. The ratio of partial pressure of oxygen in arterial blood to the fractional of inspiratory oxygen concentration in COVID-19-induced ARDS was lower than that in influenza A induced ARDS during the initial stages of mechanical ventilation (influenza A induced ARDS 216.1 vs. COVID-19-induced ARDS 167.9, *p* = 0.009, day 1). The positive end expiratory pressure remained consistently higher in the COVID-19 group throughout the follow-up period (7.0 vs. 10.0, *p* < 0.001, day 1). COVID-19 and influenza A initially showed different directions for peak inspiratory pressure and dynamic compliance; however, after day 3, both groups exhibited similar directions. Dynamic driving pressure exhibited opposite trends between the two groups during mechanical ventilation.

**Conclusions:**

Respiratory mechanics show clear differences between COVID-19-induced ARDS and influenza A induced ARDS. Based on these findings, we can consider future treatment strategies for COVID-19-induced ARDS.

## Background

Acute respiratory distress syndrome (ARDS) is characterized by bilateral pulmonary edema caused by increased permeability of the alveolar capillary membrane, severe hypoxemia, and reduced lung compliance [1, 2]. During the recent COVID-19 pandemic, COVID-19-induced ARDS emerged as a significant cause of death among patients, and its mortality rate is comparable to that of conventional ARDS [3, 4].

Moreover, comparative studies found histopathological and clinical differences between influenza A induced ARDS and COVID-19-induced ARDS, both of which share common features of virus-induced ARDS [5–8]. However, research on whether there are differences in respiratory mechanics during mechanical ventilation between the two diseases is lacking. Furthermore, while the adjustment of mechanical ventilation is a crucial factor in the treatment of ARDS, additional research is necessary to determine whether there are differences in the methods of mechanical ventilation adjustment between the two diseases.

Various perspectives have emerged regarding the treatment of COVID-19-induced ARDS. Given that the respiratory biomechanical characteristics of COVID-19-induced ARDS closely resemble those of other forms of ARDS, some stances advocate for the application of traditional ARDS treatment protocols [9]. Conversely, others argue that the distinctive respiratory dynamics evident at different stages of COVID-19-induced ARDS should be acknowledged, urging consideration of alternative approaches to treatment for each stage [10].

Therefore, this study aimed to ascertain whether there is a difference in respiratory mechanics during mechanical ventilation over time between COVID-19-induced ARDS and influenza A induced ARDS, and to determine whether a different approach for controlling mechanical ventilation is necessary in the future for these diseases.

## Methods

### Study design and population

This single-center, retrospective, observational cohort study was conducted at a tertiary referral hospital in the Republic of Korea. The study included intensive care unit (ICU) patients aged ≥ 19 years old who were diagnosed with ARDS according to the Berlin definition among patients with COVID-19 or Influenza A between January 2015 and July 2021. Of the 92 patients registered in the cohort, five who underwent extracorporeal membrane oxygenation (ECMO), which could potentially impact mechanical ventilation settings, were excluded. Finally, 42 and 45 patients with COVID-19-induced ARDS and Influenza A induced ARDS, respectively, were included.

### Collection and definition of variables

Demographic data and Charlson comorbidity index (CCI) scores were collected upon ICU admission. The predicted body weight (PBW) was calculated based on sex, with the formula 50 + 0.91 × (height (cm)–152.4) for males, and 45.5 + 0.91 × (height (cm)–152.4) for females [11]. The Sequential Organ Failure Assessment (SOFA) score was determined based on the patient’s condition at the time of intubation. The use of vasopressors and renal replacement therapy (RRT) was indicated if they occurred within seven days after intubation and lasted for at least one day. Weaning success was defined as successful extubation and absence of ventilator support within 48 h.

The diagnosis of COVID-19 and Influenza A was confirmed when the first positive result was obtained during hospitalization using a reverse transcription-polymerase chain reaction assay of respiratory samples. The diagnosis of ARDS was confirmed if the criteria outlined in the 2012 Berlin definition for timing, chest imaging, and origin of edema were met, and if the ratio of partial pressure of oxygen in arterial blood to the fractional of inspiratory oxygen concentration (P/F ratio) on the day of intubation (Day 0) indicated at least mild oxygenation impairment (P/F ratio ≤ 300 mmHg with positive end expiratory pressure [PEEP] or continuous positive airway pressure [CPAP] ≥ 5 cmH_2_O) as per the oxygenation category [1].

### Collection and definition of ventilatory parameters

Ventilation parameters for respiratory mechanics were collected at specific times on days one, three, and seven after intubation. The collected variables were exhaled minute volume (Mve), respiratory rate, tidal volume (TV), peak inspiratory pressure (PIP), PEEP, fraction of inspired oxygen (FiO2), and in the case of the pressure support mode, it was the pressure support pressure. The dynamic driving pressure (DP) and dynamic compliance (Cdyn) were estimated using the following equations: DP = PIP–PEEP, and Cdyn = TV/(PIP–PEEP), respectively.

### Treatment and management of ARDS

To maximize the implementation of a protective ventilation approach, TV was set to ≤ 7 ml/kg of PBW and PIP was set to ≤ 30 cm H2O immediately after intubation. A minimal level of sedation and analgesia necessary to achieve synchronization with the mechanical ventilator was administered, and stress ulcer prophylaxis and deep venous thrombosis prophylaxis were applied unless contraindicated [2]. During hospitalization, if additional bacterial infections occurred in both disease groups, appropriate antibiotics were administered based on the culture results, and the recommended nutrition was provided to both disease groups via consultation with a nutrition specialist. A spontaneous breathing trial was conducted when the ventilator parameters allowed for FiO_2_ ≤ 0.35 and PEEP ≤ 5 cmH2O, as part of the weaning process. Patients with ARDS diagnosed with influenza A received antiviral drugs such as oseltamivir or peramivir without steroid treatment, whereas those diagnosed with COVID-19 were administered steroids [4, 7].

### Statistical analysis

The normality of the data was assessed using the Shapiro–Wilk test. Pearson’s chi-squared test or Fisher’s exact test was applied for categorical variables and Mann–Whitney U test was used for continuous variables. Continuous variables were presented as medians (interquartile range) because most variable distributions were non-normal distribution. Statistical significance was set at *p* < 0.05. R software (v.4.2.1, Vienna, Austria) and SPSS (v.26.0, New York, USA) were used for all statistical analyses.

## Results

### Baseline characteristics

The baseline characteristics of the 87 patients are presented in Table 1. Among the total patients, 63.2% were male, and the median age was 71.0 (62.0–78.0) years old. The influenza A induced ARDS group had higher CCI and SOFA scores at intubation, along with higher rates of vasopressor and RRT use, and mortality than the COVID-19-induced ARDS group. Tracheostomy and weaning success rates did not differ significantly between the groups; however, the influenza A induced ARDS group had a shorter ICU stay than the COVID-19-induced ARDS group (Influenza A induced ARDS 10.0 days vs. COVID-19-induced ARDS 24.5 days, *p* < 0.001).

**Table 1:** Baseline characteristics between influenza A induced ARDS and COVID-19-induced ARDS.

Characteristics	Total participants (*N* = 87)	Influenza A induced ARDS (*N* = 45)	COVID-19-induced ARDS (*N* = 42)	p-value
Age (year)	71.0 (62.0–78.0)	73.0 (62.5–79.5)	69.5 (62.0–76.0)	0.322
Male, no. (%)	55 (63.2)	29 (64.4)	26 (61.9)	0.806
Smoking status (Current or former), no. (%)	25 (28.7)	14 (31.1)	11 (26.2)	0.612
BMI (kg/m^2^)	22.8 (19.9–25.1)	20.3 (17.7–24.1)	24.3 (22.2–25.9)	< 0.001
CCI	1.0 (0.0–4.0)	3.0 (1.0–5.0)	1.0 (0.0–1.3)	< 0.001
SOFA	9.0 (5.0–13.0)	11.0 (7.5–14.5)	6.5 (4.0–11.0)	< 0.001
Use of vasopressor, no (%)	71 (81.6)	42 (93.3)	29 (69.0)	0.005
RRT, no (%)	13 (14.9)	12 (26.7)	1 (2.4)	0.002
ICU hospitalization period (day)	20.0 (9.0–32.0)	10.0 (7.0–23.5)	24.5 (19.0–51.5)	< 0.001
28-day mortality, no (%)	20 (23.0)	16 (35.6)	4 (9.5)	0.005
Final mortality, no (%)	32 (36.8)	22 (48.9)	10 (23.8)	0.015
Tracheostomy, no (%)	20 (23.0)	8 (17.8)	12 (28.6)	0.232
Weaning success, no (%)	49 (56.3)	25 (55.6)	24 (57.1)	0.881

Data are presented as no. (%) or median (IQR)

ARDS, acute respiratory distress syndrome; BMI, body mass index; CCI, Charlson comorbidity index; SOFA, sequential organ failure assessment score; RRT, Renal replacement therapy; ICU, intensive care unit

### Differences in respiratory mechanics during mechanical ventilation

**Fig. 1:**
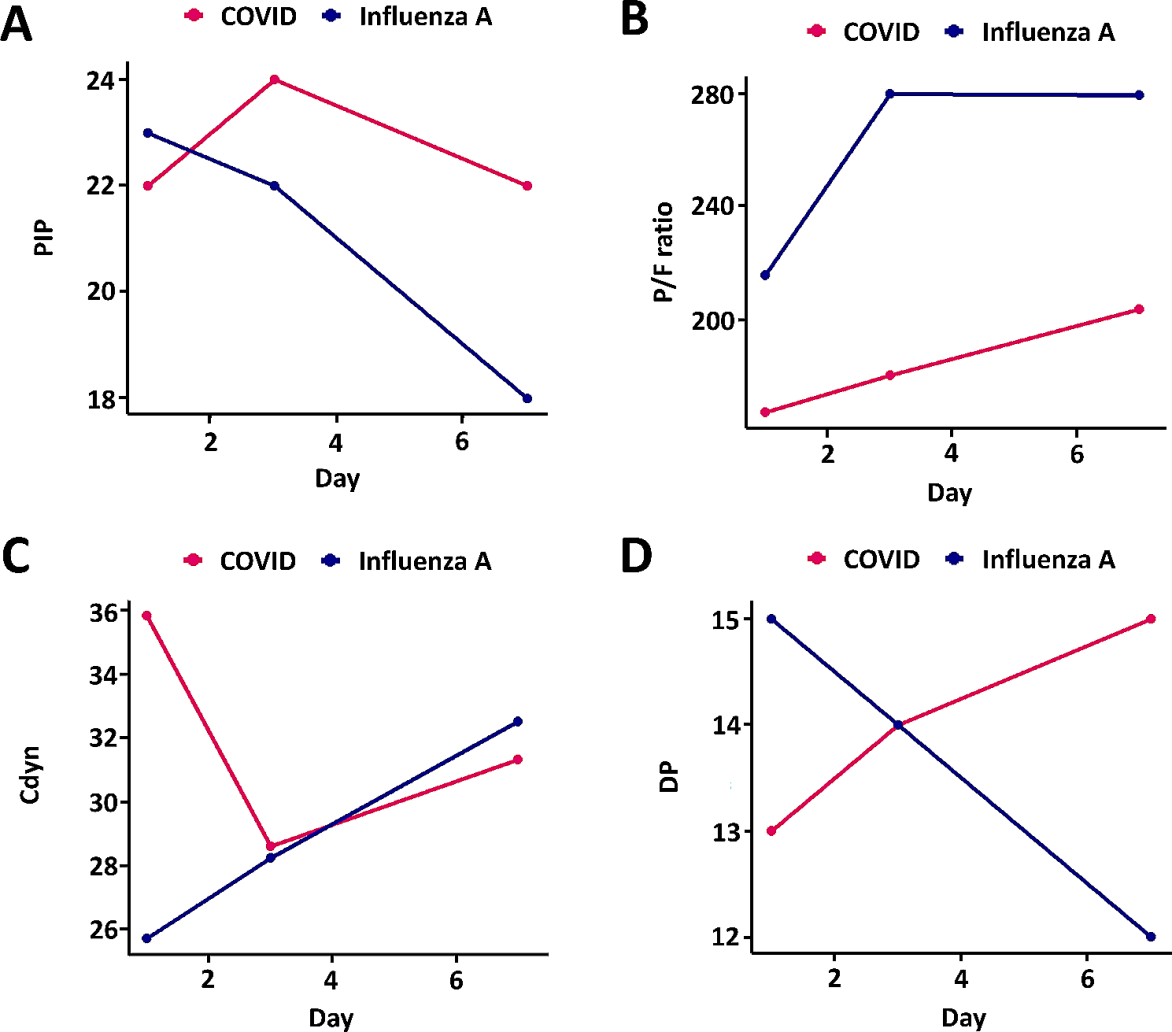
Changes in respiratory mechanics between COVID-19-induced ARDS and influenza A induced ARDS. Median values of each parameter, including PIP (A), P/F ratio (B), Cdyn (C), and DP (D), are displayed according to the number of days after intubation. PIP, peak inspiratory pressure; P/F ratio, arterial partial pressure of oxygen (PaO_2_) divided by inspired oxygen concentration (FiO_2_); Cdyn, dynamic compliance; DP, dynamic driving pressure

Table 2 presents the respiratory mechanics differences over time after intubation between COVID-19-induced ARDS and Influenza A induced ARDS. On day 1, the Influenza A group had a higher P/F ratio (216.1 vs. 167.9, *p* = 0.009) and lower PEEP (8.0 cm H_2_O vs. 10.0 cm H_2_O, *p* < 0.001) than the COVID-19 group, however, they showed lower Cdyn (25.7 cm H_2_O vs. 35.8 cm H_2_O, *p* = 0.001). Starting from day 3, the P/F ratio showed a statistically significant difference like the 1st day (279.5 vs. 181.1, *p* = 0.009), but there were no statistical differences in Cdyn and DP between the two groups. PEEP remained consistently higher in the COVID-19 group throughout the follow-up period. A schematic diagram of this is illustrated in Fig. 1. The overall trend is that the COVID-19 and Influenza A groups exhibit similar increasing patterns in P/F ratio over time. In the case of pip and Cdyn, the directions of COVID and influenza A were initially different. However, after day 3, both groups exhibited a decrease in PIP and an increase in Cdyn. Unlike other indicators, DP exhibited opposite trends, increasing consistently in the COVID-19 group and decreasing consistently in the Influenza A group.

**Table 2:** Differences in respiratory mechanics during mechanical ventilation between influenza A- and COVID-19-induced ARDS.

**Day 1**				
**Characteristics**	**Total participants** (**N = 87)**	**Influenza A induced ARDS** (**N = 45)**	**COVID-19-induced ARDS** (**N = 42)**	**p-value**
Pco_2_	38.9 (33.5–43.6)	37.5 (33.8–44.3)	40.0 (33.0–43.3)	0.584
Pa_O2_:Fi_O2_	184.7 (135.7–243.6)	216.1 (134.2–271.3)	167.9 (137.5–212.7)	0.009
Mve (L/min)	8.9 (7.6–10.3)	8.5 (7.2–10.2)	9.0 (8.0–10.3)	0.707
TV/PBW (ml/kg)	7.2 (6.3–8.4)	7.0 (6.3–8.3)	7.2 (6.3–8.5)	0.763
PIP (cm H_2_O)	23.0 (20.0–26.0)	23.0 (20.5–27.0)	22.0 (19.0–25.0)	< 0.001
PEEP (cm H_2_O)	8.0 (7.0–10.0)	8.0 (5.0–8.0)	10.0 (8.0–12.0)	< 0.001
Cdyn (cm H_2_O) ^a^	30.0 (23.7–37.9)	25.7 (21.3–34.4)	35.8 (28.9–41.6)	0.001
DP (cm H_2_O) ^b^	14.0 (12.0–17.0)	15.0 (13.5–19.0)	13.0 (9.8–16.0)	0.369
**Day 3**				
**Characteristics**	**Total participants** **(N = 80)**	**Influenza A induced ARDS ** **(N = 39)**	**COVID-19-induced ARDS** **(N = 41)**	**p-value**
Pco_2_	38.8 (33.3–44.2)	37.7 (30.3–47.1)	39.8 (35.0–42.8)	0.763
Pa_O2_:Fi_O2_	215.2 (158.2–309.0)	279.5 (172.0–333.0)	181.0 (141.0–252.0)	0.005
Mve (L/min)	8.4 (7.3–9.8)	7.9 (6.1–9.3)	8.4 (7.7–9.9)	0.072
TV/PBW (ml/kg)	7.3 (6.5–7.8)	7.2 (6.4–8.0)	7.3 (6.6–7.8)	0.969
PIP (cm H_2_O)	23.0 (18.3–25.8)	22.0 (16.0–25.0)	24.0 (20.0–26.5)	0.051
PEEP (cm H_2_O)	8.0 (6.0–10.0)	6.0 (5.0–8.0)	10.0 (8.0–11.0)	< 0.001
Cdyn (cm H_2_O) ^a^	28.4 (22.7–39.0)	28.2 (21.4–47.9)	28.6 (23.1–37.9)	0.935
DP (cm H_2_O) ^b^	14.0 (11.0–17.0)	14.0 (9.0–18.0)	14.0 (12.0–17.0)	0.817
**Day 7**				
**Characteristics**	**Total participants** **(N = 64)**	**Influenza A induced ARDS ** **(N = 27)**	**COVID-19-induced ARDS** **(N = 37)**	**p-value**
Pco_2_	38.2 (34.0–42.5)	36.9 (31.6–42.6)	39.1 (35.1–42.6)	0.799
P/F ratio	215.0 (146.4–310.7)	279.1 (143.6–353.3)	204.2 (147.6–282.5)	0.170
Mve (L/min)	8.8 (7.5–10.6)	8.6 (6.4–10.1)	8.9 (8.1–10.9)	0.123
TV/PBW (ml/kg)	7.4 (6.5–8.5)	7.4 (6.0–8.7)	7.4 (6.6–8.4)	0.838
PIP (cm H_2_O)	21.0 (16.3–25.0)	18.0 (13.0–25.0)	22.0 (19.0–25.5)	0.108
PEEP (cm H_2_O)	8.0 (5.0–10.0)	6.0 (5.0–7.0)	8.0 (8.0–10.0)	< 0.001
Cdyn (cm H_2_O) ^a^	31.3 (23.7–45.3)	32.5 (20.5–62.2)	31.3 (24.5–37.8)	0.951
DP (cm H_2_O) ^b^	13.5 (9.3–16.8)	12.0 (8.0–18.0)	15.0 (11.0–16.0)	0.643

Data are presented as no. (%) or median (IQR)

Pco_2,_ partial pressure of carbon dioxide; P/F ratio, the arterial partial pressure of oxygen (PaO2) divided by the inspired oxygen concentration (FiO2); Mve, Exhaled minute volume; TV, tidal volume; PBW, predicted body weight, PIP, peak inspiratory pressure; PEEP, positive end expiratory pressure; Cdyn, dynamic compliance; DP, dynamic driving pressure

^a^ Cdyn = TV/(PIP-PEEP)

^b^ DP = PIP-PEEP

### Differences in respiratory mechanics during mechanical ventilation between COVID-19-induced ARDS and Influenza A induced ARDS survivors

Table 3 compares the baseline characteristics of COVID-19-induced ARDS and influenza A induced ARDS in survivors. Influenza A induced ARDS survivors had lower BMI (19.9 kg/m^2^ vs. 24.1 kg/m^2^, *p* < 0.001) and higher CCI (3.0 vs. 0.0, *p* < 0.001) and SOFA scores (10.0 vs. 5.0, *p* < 0.001) compared to COVID-19-induced ARDS survivors. However, tracheostomy and weaning success rates were similar between the two groups, and the ICU hospitalization period was shorter in influenza A induced ARDS survivors (9.0 days vs. 22.0 days, *p* < 0.001). Additionally, there were no statistically significant differences in the use of vasopressors or RRT in the survivor group, unlike in overall participants.

**Table 3:** Baseline characteristics between influenza A induced ARDS and COVID-19-induced ARDS survivors

Characteristics	Total participants (*N* = 55)	Influenza A induced ARDS (*N* = 23)	COVID-19-induced ARDS (*N* = 32)	p-value
Age (year)	70.0 (59.0–75.0)	70.0 (59.0–78.0)	69.0 (59.8–74.8)	0.322
Male, no. (%)	30 (54.5)	12 (52.2)	18 (56.3)	0.765
Smoking status (Current or former), no. (%)	12 (21.8)	4 (17.4)	8 (25.0)	0.742
BMI (kg/m^2^)	22.6 (19.7–25.1)	19.9 (16.9–22.5)	24.1 (22.2–25.5)	< 0.001
CCI	1.0 (0.0–3.0)	3.0 (1.0–5.0)	0.0 (0.0–1.0)	< 0.001
SOFA	7.0 (4.0–12.0)	10.0 (5.0–12.0)	5.0 (3.3–9.0)	< 0.001
Use of vasopressor, no (%)	40 (72.7)	20 (87.0)	20 (62.5)	0.066
RRT, no (%)	3 (5.5)	3 (13.0)	0 (0.0)	0.068
ICU hospitalization period (day)	19.0 (10.0–29.0)	9.0 (7.0–16.0)	22.0 (19.0–42.3)	< 0.001
Tracheostomy, no (%)	12 (21.8)	6 (26.1)	6 (18.8)	0.516
Weaning success, no (%)	46 (83.6)	22 (95.7)	24 (75.0)	0.064

Data are presented as no. (%) or median (IQR)

ARDS, acute respiratory distress syndrome; BMI, body mass index; CCI, Charlson comorbidity index; SOFA, sequential organ failure assessment score; RRT, Renal replacement therapy; ICU, intensive care unit

**Fig. 2:**
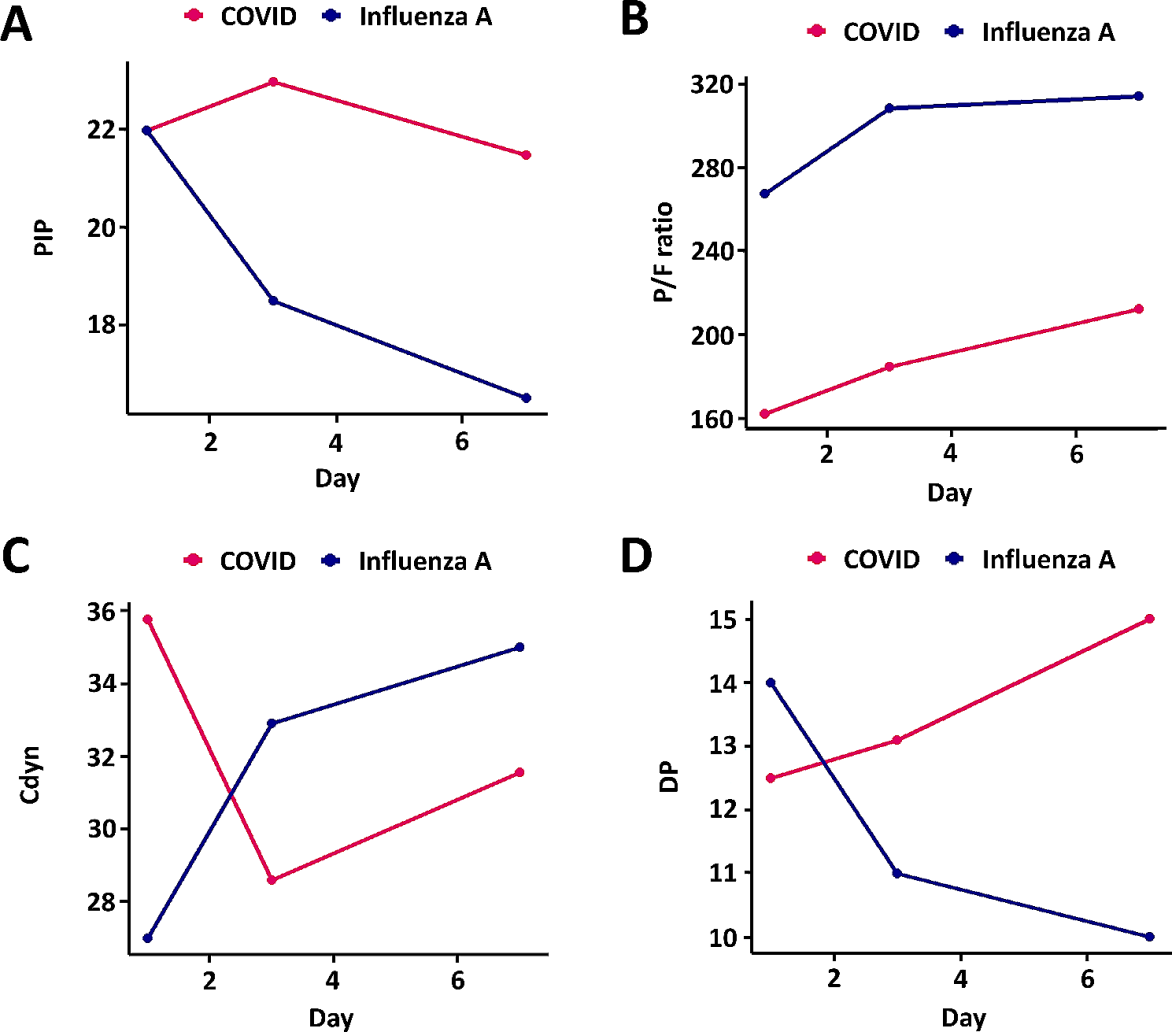
Changes in respiratory mechanics between COVID-19-induced ARDS and influenza A induced ARDS survivors. Median values of each parameter, including PIP (A), P/F ratio (B), Cdyn (C), and DP (D), are displayed according to the number of days after intubation. PIP, peak inspiratory pressure; P/F ratio, arterial partial pressure of oxygen (PaO_2_) divided by inspired oxygen concentration (FiO_2_); Cdyn, dynamic compliance; DP, dynamic driving pressure

Table 4 presents changes in respiratory mechanics over time during mechanical ventilation in survivors. On Day 1, COVID-19-induced ARDS survivors had a lower P/F ratio (268.0 vs. 162.5, *p* < 0.001) and higher PEEP (7.0 cm H_2_O vs. 10.0 cm H_2_O, *p* < 0.001) compared to Influenza A induced ARDS survivors, which was consistent with the results in the overall participants. However, there were no significant differences in PIP and Cdyn, unlike the entire patient population, among the survivor groups. On Day 3 and Day 7, COVID-19-induced ARDS survivors had higher PIP (18.5 cm H_2_O vs. 23.0 cm H_2_O, *p* = 0.001), and DP (11.0 cm H_2_O vs. 13.1 cm H_2_O, *p* = 0.044). Throughout the study, differences in P/F ratio, Mve, and PEEP between Influenza A induced ARDS survivors and COVID-19-induced ARDS survivors persisted. The graphical representation of respiratory mechanics over time in Fig. 2 closely paralleled the results of Fig. 1 for the entire patient group.

**Table 4:** Differences in respiratory mechanics during mechanical ventilation between influenza A- and COVID-19-induced ARDS survivors

**Day 1**				
**Characteristics**	**Total participants** (**N = 55)**	Influenza A induced ARDS (**N = 23)**	COVID-19-induced ARDS (**N = 32)**	**p-value**
Pco_2_	38.0 (35.1–43.6)	37.1 (34.4–40.5)	40.3 (36.8–44.3)	0.096
Pa_O2_:Fi_O2_	194.4 (149.4–268.3)	268.0 (196.4–304.3)	162.5 (139.4–208.3)	< 0.001
Mve (L/min)	8.7 (7.2–10.3)	7.8 (6.1–8.5)	9.4 (8.0–10.4)	0.005
TV/PBW (ml/kg)	7.2 (6.6–7.8)	7.0 (6.6–8.0)	7.2 (6.4–7.8)	0.824
PIP (cm H_2_O)	22.0 (19.0–25.0)	22.0 (19.0–25.0)	22.0 (19.0–25.0)	0.537
PEEP (cm H_2_O)	8.0 (7.0–10.0)	7.0 (5.0–8.0)	10.0 (8.0–11.8)	< 0.001
Cdyn (cm H_2_O) ^a^	33.4 (25.6–40.0)	27.0 (23.5–36.8)	35.7 (28.7–42.9)	0.056
DP (cm H_2_O) ^b^	14.0 (11.0–16.0)	14.0 (12.0–17.0)	12.5 (9.3–15.8)	0.072
**Day 3**				
**Characteristics**	**Total participants** **(N = 53)**	**Influenza A induced ARDS ** **(N = 22)**	**COVID-19-induced ARDS** **(N = 31)**	**p-value**
Pco_2_	38.9 (33.7–42.6)	36.4 (32.3–41.8)	40.0 (35.1–42.6)	0.136
Pa_O2_:Fi_O2_	269.3 (171.3–333.0)	308.9 (276.9–379.1)	185.0 (157.0–275.6)	< 0.001
Mve (L/min)	8.3 (7.2–9.8)	7.2 (5.5–9.2)	8.7 (7.7–9.9)	0.012
TV/PBW (ml/kg)	7.3 (6.5–7.8)	7.4 (6.3–8.0)	7.3 (6.5–7.8)	0.691
PIP (cm H_2_O)	21.0 (17.5–24.5)	18.5 (14.5–22.0)	23.0 (20.0–25.0)	0.001
PEEP (cm H_2_O)	8.0 (6.0–10.0)	6.0 (5.0–8.0)	10.0 (8.0–10.0)	< 0.001
Cdyn (cm H_2_O) ^a^	30.2 (25.0–41.6)	32.9 (27.3–56.1)	28.6 (22.7–38.9)	0.108
DP (cm H_2_O) ^b^	13.0 (10.5–16.0)	11.0 (8.0–15.3)	13.1 (12.0–17.0)	0.044
**Day 7**				
**Characteristics**	**Total participants** **(N = 42)**	**Influenza A induced ARDS ** **(N = 14)**	**COVID-19-induced ARDS** **(N = 28)**	**p-value**
Pco_2_	36.7 (33.5–41.6)	35.5 (29.1–39.1)	37.3 (34.0–42.2)	0.196
P/F ratio	260.3 (182.2–336.9)	314.6 (267.9–366.8)	212.9 (174.2–283.9)	0.014
Mve (L/min)	8.6 (6.9–10.7)	6.7 (5.7–8.8)	9.3 (8.3–11.1)	0.001
TV/PBW (ml/kg)	7.6 (6.6–8.6)	7.5 (6.0–9.1)	7.6 (6.8–8.6)	0.626
PIP (cm H_2_O)	20.0 (14.8–24.0)	16.5 (12.8–20.0)	21.5 (17.3–24.8)	0.007
PEEP (cm H_2_O)	8.0 (5.0–8.3)	6.0 (5.0–7.0)	8.0 (7.0–10.0)	0.005
Cdyn (cm H_2_O) ^a^	32.1 (27.3–55.3)	35.0 (28.0–81.6)	31.5 (26.1–38.6)	0.348
DP (cm H_2_O) ^b^	13.0 (8.0–16.0)	10.0 (7.0–13.3)	15.0 (10.3–16.0)	0.048

Data are presented as no. (%) or median (IQR)

Pco_2,_ partial pressure of carbon dioxide; P/F ratio, the arterial partial pressure of oxygen (PaO2) divided by the inspired oxygen concentration (FiO2); Mve, Exhaled minute volume; TV, tidal volume; PBW, predicted body weight, PIP, peak inspiratory pressure; PEEP, positive end expiratory pressure; Cdyn, dynamic compliance; DP, dynamic driving pressure

^a^ Cdyn = TV/(PIP-PEEP)

^b^ DP = PIP-PEEP

## Discussion

We compared COVID-19-induced ARDS and influenza A induced ARDS, a representative condition of virus-induced ARDS, to investigate whether there were differences in respiratory mechanics during mechanical ventilation. In the initial phases of mechanical ventilation, disparities in respiratory mechanics were observed between the two groups, persisting in some variables over time and diminishing in others.

This difference was maintained with respect to hypoxemia. Our results revealed that the P/F ratio in patients with COVID-19-induced ARDS was significantly lower than that in patients with influenza A induced ARDS during the early stages of mechanical ventilation in all participants. Nevertheless, contradictory results have been observed in previous studies with respect to the P/F ratio. Cobb et al. reported no significant difference between the two disease groups on mechanical ventilation day 1; however, from day 2 onwards, the P/F ratio in the COVID-19 group was significantly lower [12]. In contrast, Tang et al. and Ding et al. showed that the P/F ratio in COVID-19-induced ARDS was higher than that in influenza A induced ARDS [13, 14]. Considering ECMO was provided to patients in the studies by Tang et al. and Ding et al., it is possible that differences in disease severity led to variations in the reduction of the P/F ratio in each condition. In our study, we analyzed data after excluding patients receiving ECMO; therefore, we speculate that a relatively higher proportion of mild ARDS cases may have resulted in findings similar to those of Cobb et al. Furthermore, PEEP exhibited higher values in both the entire group and the group of survivors among COVID-19 patients, which is surmised to be a clinical response aimed at compensating for the low P/F ratio. The lower P/F ratio in COVID-19-induced ARDS may be attributed to relatively increased microthrombosis and endothelialitis compared with influenza A patients, potentially leading to a ventilation-perfusion mismatch and disruption of pulmonary vasoregulation [10, 15]. Additionally, histopathological studies showing a higher incidence of microthrombosis in COVID-19 patients than in influenza A patients, along with spatial transcriptomic studies demonstrating greater expression of coagulation-related genes in COVID-19 than in influenza A, support this finding [5, 6].

In our results, the difference in lung compliance between the two diseases diminished. In the case of COVID-19-induced ARDS, initial compliance was significantly higher than that of influenza A induced ARDS. However, over time, Cdyn in COVID-19 decreased relatively quickly, and showed no statistical difference compared with the influenza A group after day 3. Previous studies have reported that lung compliance is higher in COVID-19-induced ARDS than in classic ARDS [15, 16]. In a study by Kronibus et al., which examined changes in compliance over time, initial compliance did not show a statistically significant difference between the two groups. However, the absolute value of median compliance in COVID-19 patients was higher, and over time, a trend was observed where the difference in median values between the two groups decreased, similar to the results of our study [17]. Therefore, in the case of COVID-19-induced ARDS, compliance is higher in the early stages than that in influenza A induced ARDS; however, over time, it becomes similar to that of influenza A induced ARDS. Moreover, Influenza A showed a time-dependent decrease in the DP, which was inversely related to Cdyn (Fig. 1D), while COVID-19 did not exhibit a complete inverse correlation with Cdyn. This is presumed to be because the increase in TV due to recovery between Days 3 and 7 was reflected in Cdyn but not in DP, and this trend was more pronounced in the analysis of survivors (Fig. 2D).

Based on our findings regarding the respiratory mechanics of COVID-19-induced ARDS, we propose the following treatment strategies. First, COVID-19-induced ARDS exhibited a relatively low P/F ratio in the early stages of endotracheal intubation. Therefore, early implementation of the prone position, a method validated in previous studies, is necessary along with simultaneously efforts to reduce hypoperfusion of well-ventilated lung areas and improve ventilation-perfusion mismatch through personalized PEEP ventilation adjustments to improve early hypoxemia [18–21]. However, the relatively high lung compliance in COVID-19-induced ARDS may lead to an overestimation of the patient’s condition, posing a risk of delayed introduction of the prone position. Thus, when assessing the severity of COVID-19-induced ARDS, clinical decision-making should primarily consider the P/F ratio. Furthermore, recent clinical practice guidelines for ARDS recommend considering the application of venovenous ECMO in cases of severe ARDS with a low P/F ratio [22, 23]. Positive impacts of ECMO on short-term survival have been also noted in observational studies of respiratory failure induced by COVID-19 [24, 25]. Therefore, early implementation of the prone position and the consideration of ECMO should also be prioritized in COVID-19-induced ARDS. Second, as time elapses following the abrupt decrease in lung compliance in the early stages, the respiratory mechanic pattern of COVID-19-induced ARDS becomes similar to influenza A induced ARDS. This emphasizes the importance of a lung protective strategy in COVID-19-induced ARDS. Influenza A induced ARDS with initially low lung compliance tends to improve over time and has a relatively low risk of barotrauma even with delayed adjustments to the ventilator. However, in case of COVID-19, which exhibits a rapid decrease in lung compliance in the initial stage, there is a higher likelihood of ventilator-induced lung injury occurring if real-time ventilator setting adjustments are insufficient due to limitations in medical resources. Therefore, the importance of low tidal volume becomes even more marked in the case of COVID-19-induced ARDS compared to that in other forms of ARDS.

According to a previous study, the median duration from the onset of symptoms to the development of ARDS was 9.5 days in the case of COVID-19, whereas it was relatively shorter at 7.0 days for influenza A [14]. The onset of ARDS appears to be more influenced by cytokine levels than by viral load, and the peak cytokine level is known to occur earlier in influenza A than in COVID-19. This may explain the difference in the ARDS onset period, as previously described [26, 27]. Owing to the difference in the timing of the cytokine peak, it is possible that in COVID-19-induced ARDS, there was a relatively high Cdyn on day 1, followed by a sharp decrease on day 3 and an increase in PIP. However, in the case of COVID-19, although the cytokines reach their peak later, they remain at elevated levels for a much longer duration. As a result, overall cumulative cytokine exposure is greater in patients with COVID-19 patients than with influenza A [27]. Hence, persistent inflammation could contribute to increased DP and elevated PIP in COVID-19-induced ARDS, leading to delays in weaning and a prolonged ICU hospitalization period. In our study, despite the relatively unfavorable clinical indicators of influenza A patients at the time of ICU admission, as predicted by a previous study, we found that the weaning outcomes between survivors were similar between the two groups, with influenza A patients showing a relatively shorter ICU hospitalization period.

This study had some limitations. First, this was a single-center study, which may have been influenced by regional variations in COVID-19 incidence rates. However, involvement of the same institution provided simultaneous benefits because it was possible to reduce the impact of time frame differences on data quality, specifically in terms of equipment, healthcare providers, and adherence to the protocol. Second, at the end of the research period in July 2021, the vaccination rate for COVID-19 was significantly different from that for influenza A. Approximately only 10% of the population in South Korea achieved full COVID-19 vaccination in July 2021 [28]. On the other hand, approximately 30–40% of South Korea’s entire population had been vaccinated against Influenza A, with an 80% vaccination rate among those aged ≥ 65 [29]. Therefore, it cannot be ruled out that patients with influenza A induced ARDS with worse clinical indicators were included, as patients who developed ARDS despite vaccination tended to have an unfavorable clinical status.

## Conclusions

The respiratory mechanics of COVID-19-induced ARDS and influenza A induced ARDS have been proven to differ significantly. The findings of this study are anticipated to form the basis for devising future treatment strategies that contemplate the optimal adjustment of ventilators at distinct stages of mechanical ventilation in patients with COVID-19-induced ARDS.

## Data Availability

The datasets used and analyzed during the present study are available from the corresponding author on reasonable request.

## Abbreviations

ARDS: Acute respiratory distress syndrome
CCI: Charlson comorbidity index
Cdyn: Dynamic compliance
CPAP: Continuous positive airway pressure
DP: Dynamic driving pressure
ECMO: Extracorporeal membrane oxygenation
FiO2: Fraction of inspired oxygen
ICU: Intensive care unit
Mve: Exhaled minute volume
P/F ratio: The ratio of partial pressure of oxygen in arterial blood to the fractional of inspiratory oxygen concentration
PBW: Predicted body weight
PEEP: Positive end expiratory pressure
PIP: Peak inspiratory pressure
RRT: Renal replacement therapy
SOFA: The sequential organ failure assessment
TV: Tidal volume
